# The monoclonal S9.6 antibody exhibits highly variable binding affinities towards different R-loop sequences

**DOI:** 10.1371/journal.pone.0178875

**Published:** 2017-06-08

**Authors:** Fabian König, Thomas Schubert, Gernot Längst

**Affiliations:** 1 Biochemistry III; Biochemistry Centre Regensburg (BCR), University of Regensburg, Universitätsstr, Regensburg, Germany; 2 2Bind GmbH, Regensburg, Germany; Florida International University, UNITED STATES

## Abstract

The monoclonal antibody S9.6 is a widely-used tool to purify, analyse and quantify R-loop structures in cells. A previous study using the surface plasmon resonance technology and a single-chain variable fragment (scFv) of S9.6 showed high affinity (0.6 nM) for DNA—RNA and also a high affinity (2.7 nM) for RNA—RNA hybrids. We used the microscale thermophoresis method allowing surface independent interaction studies and electromobility shift assays to evaluate additional RNA-DNA hybrid sequences and to quantify the binding affinities of the S9.6 antibody with respect to distinct sequences and their GC-content. Our results confirm high affinity binding to previously analysed sequences, but reveals that binding affinities are highly sequence specific. Our study presents R-loop sequences that independent of GC-content and in different sequence variations exhibit either no binding, binding affinities in the micromolar range and as well high affinity binding in the nanomolar range. Our study questions the usefulness of the S9.6 antibody in the quantitative analysis of R-loop sequences *in vivo*.

## Introduction

R-loops are local RNA-DNA hybrid sequences, generally formed by a nascent G-rich transcript hybridizing with the DNA template strand and thereby leaving the non-template DNA single stranded [1]. These structures were first described *in vitro* in 1976 and about 20 years ago in prokaryotes having a mutation in the Topoisomerase I gene [2]. R-loops were initially considered as a by-product of transcription, but during the past decade very important functions of R-loops in transcription, genomic stability and a variety of diseases emerged [3]. The persistence of R-loops can result in the accumulation of DNA double-strand breaks (DSBs) [4], leading to DNA rearrangements and genome instability [1,5].

R-loops occur naturally during transcription and serve for example in class switch recombination of immunoglobulin (Ig) genes in activated B cells [6] and are functional structures in mitochondrial DNA replication [7,8]. Genome-wide mapping techniques were established to determine R-loop occurrence in human, mouse, and yeast cells, revealing that R-loops are highly abundant, with 5% of mammalian genomic sequences and 8% of the budding yeast sequences forming R-loops [9,10]. Potential regulatory functions of these structures are implied, as R-loop sequences are frequently identified at GC-rich regions such as many promoters and 3′end regions, where they appear to play significant roles in transcription [9,11–13]. R-loops can now be effectively mapped with high-throughput methods that are based on the specific recognition of RNA-DNA hybrids by the S9.6 antibody [14,15]. The antibody was recently used to detect and localize DNA—RNA hybrids that have been linked to genomic instability, at CpG island promoters, terminator regions and genomic regions with altered chromatin structure [16–19] [9,20].

The monoclonal antibody S9.6 was originally generated in mice using an *in vitro* synthesized ΦX174 DNA—RNA antigen and shown to exhibit high specificity and affinity for DNA—RNA hybrids [14]. The antibody was initially used in assays to detect and quantify specific RNA-DNA hybrids [21–23] and for genome wide array based hybridization mapping techniques [24,25]. The specific recognition of miRNA-DNA hybrids with a length of 22nt was also used to develop sensitive biosensor systems [26,27].

Because of the widespread use of the S9.6 antibodies in research and the importance to interpret the specific binding events, a recent study sought to further characterize the binding affinities and specificity of the single-chain variable fragment (scFv) of S9.6 [15]. Surface Plasmon Resonance (SPR) experiments revealed a high binding affinity of 0.6 nM for DNA-RNA hybrids and in addition an about 5 times lower and still high binding affinity for RNA-RNA hybrids. The smallest epitope recognized by the antibody was shown to consist of 6 base pairs [15]. In contrast, genome wide hybridisation mapping techniques suggest a minimal binding length of about 15 bp, which exhibits half of the binding affinity when compared to 60 bp long RNA-DNA hybrids [25].

Since RNA-RNA duplexes form an A-helix structure that deviates from the RNA-DNA duplex structure [28], we suggest that the S9.6 antibody does not recognize the R-loop structure independent of R-loop sequence. To test this hypothesis, we used microscale thermophoresis (MST) and electromobility shift assays (EMSA) as “in solution” methods, in contrast to SPR, to determine binding affinities. Indeed, our results do suggest that the binding affinity of the S9.6 antibody varies with R-loop sequences, independent of the GC-content, revealing many sequence variants with no, or low binding affinities.

## Materials and methods

### Synthesis of nucleic acid hybrids

DNA and RNA oligonuclotides were synthesized by Sigma-Aldrich (Germany) and hybrid RNA-DNA oligonucleotides were synthesized by Integrated DNA Technologies (Coralville, IA, USA). All hybrids were synthesized with 5’ Cy3, Cy5 or FAM fluorescence labels. To prepare RNA-DNA hybrids, the oligonucleotides were mixed in equimolar ratios in Annealing Buffer (80 mM NaCl; 10 mMTris, pH 7.6, 1.5 mM MgCl_2_) heated to 95°C for 3 minutes and then slowly cooled down (10 min) to room temperature. Oligonucleotides were used in microscale thermophoresis (MST) and electromobility shift assays (EMSA) at concentrations ranging from 1 nM to 40 nM, depending on the binding affinity and Nanotemper device used for MST analysis.

### Microscale thermophoresis

MST experiments were performed with the Microscale Thermophoresis instruments Monolith NT.115 and Monolith NT.115pico (NanoTemper Technologies, Munich, Germany), using the Monolith NT^™^ capillaries (Standard treated, NanoTemper Technologies, Munich, Germany). The binding assays were performed as biological duplicates at 3–30% LED (light-emitting diode) power and measuring twice at 20, 40 and 80% MST power at a fixed temperature of 25°C. The recorded MST signal of each interaction was normalized to the same baseline fluorescence and plotted against the concentration into one graph using KaleidaGraph 4.1.

MST-binding experiments were carried out with 1 to 40 nM of fluorescently labelled R-loop Oligonucleotides. The S9.6 antibody, a purified immunoglobulin G subclass 2a of a hybridoma culture, was a generous gift of Dr. Stefan Hamperl (Stanford University). The MST reaction buffer and antibody dilutions were performed in MST-buffer (50mM Tris-HCl, pH7.6, 150mM NaCl, 10mM MgCl_2_, 0,05% Tween-20). The data were fitted with the help of the quadratic fitting formula (Kd formula) derived from the law of mass action.

### Electromobility shift assays

Electromobility shift assays (EMSAs) with a combination of differently fluorescently labelled R-loop oligonucleotides and increasing concentrations of the S9.6 antibody was performed in 20μl reaction volume of Annealing buffer (10 mM Tris pH7.6, 1mM EDTA, 100 mM KCl, 1.5 mM MgCl_2_) or MST-buffer (50mM Tris-HCl, pH7.6, 150mM NaCl, 10mM MgCl_2_, 0,05% Tween-20) supplemented with 200 ng/μl bovine serum albumin. The antibody was incubated with the R-loops for 30 min at room temperature to allow complex formation. Reactions were loaded onto 8% PAA gels in 0.4 TBE, separated at 10V/cm and the DNA was visualized by fluorescence scanning using the Typhoon FLA 9500 Instrument (GE Healthcare Life Sciences, Munich, Germany).

## Results

To test the sequence specificity of S9.6 antibody binding to R-loops, we designed a set of oligonucleotides, with varying GC-content and length (Table 1). In order to evaluate the quality and specificity of our assays we did also include the 23GC52L sequence (23 nucleotide in length, 52% GC-content, Oligos are linked by a loop of four Thymidin residues) that was previously used by Phillips and colleagues [15]. The Table 1 summarizes the sequences and the binding affinities determined by MST (this study), EMSA (this study), SPR and titration experiments [14,15].

**Table 1:** Overview to the experimental results obtained by this study and comparable studies. The name of the molecules, their sequence and their binding affinities towards the S9.6 antibody determined by microscale thermophoresis assays and EMSA are given.

Molecule	Sequence	MST	EMSA	other
D23GC17L	dAdAdTdTdAdCdAdTdTdGdAdTdAdGdAdAdTdTdAdTdTdAdG-TTTT-	n.b.	n.b	
	dTdTdAdAdTdGdTdAdAdCdTdAdTdCdTdTdAdAdTdAdAdTdC			
23GC52L	dCdGdGdTdGdTdGdGdTdCdGdCdTdGdTdAdAdTdCdAdGdAdA-TTTT-	3.1 (±1) nM	20 nM	0.47 (±.08) nM [1]
	rGrCrCrArCrArCrCrArGrCrGrArCrArUrUrArGrUrCrUrU			
29GC21	DNA strand: AGAAAAAAAAAAAAAAAGAAAAAAGGAGG	n.b.	n.b.	
	RNA strand: UCUUUUUUUUUUUUUUUCUUUUUUCCUCC			
23GC52	DNA strand: CGGTGTGGTCGCTGTAATCAGAA	5.7 (±2.2) nM	40 nM	
	RNA strand: GCCACACCAGCGACAUUAGUCUU			
22GC10	DNA strand: CGGTGTGGTCGCTGTAATCAGAA	b.i.	100 nM	
	RNA strand: GCCACACCAGCGACAUUAGUCUU			
22GC75	DNA strand: CTCACCCGCCGGACCCCTCT	ca. 1500 nM	>1000 nM	
	RNA strand: GAGUGGGCGGCCUGGGGAGA			
22GC90	DNA strand: GGACGGCGGCGGCTGCGGGC	b.i.	128 nM	
	RNA strand: CCUGCCGCCGCCGACGCCCG			
15GC0A	DNA strand: TTTTTTTTTTTTTTT	>650 nM	1200 nM	ca. 300 nM [15]
	RNA strand: AAAAAAAAAAAAAAA			
16GC80	DNA strand: TTTTTTTTTTTTTTT	>2900 nM	320 nM	
	RNA strand: AAAAAAAAAAAAAAA			
15GC0U	DNA strand: AAAAAAAAAAAAAAA	n.b	n.b.	
	RNA strand: UUUUUUUUUUUUUUU			

n.b.: no binding. b.i.: binding indicated

MicroScale Thermophoresis (MST) represents a powerful technology to quantify the affinities of protein-nucleic acid interactions in solution, requiring only low amounts of the potential binding partners. The assay is based on the directed movement of molecules along a temperature gradient, relative to their thermophoretic properties [29,30]. A small and local temperature difference ΔT, induced by an infrared laser, results in a depletion of the molecules in the region of elevated temperature. The Soret coefficient ST: chot/ccold = exp(-ST ΔT) provides a quantitative measure for this effect and is depending on the size, charge and hydration shell of the molecules. Upon molecular interaction, at least one of these parameters is changed, resulting in distinct thermophoretic movements of the unbound and bound states [31].

**Fig 1:**
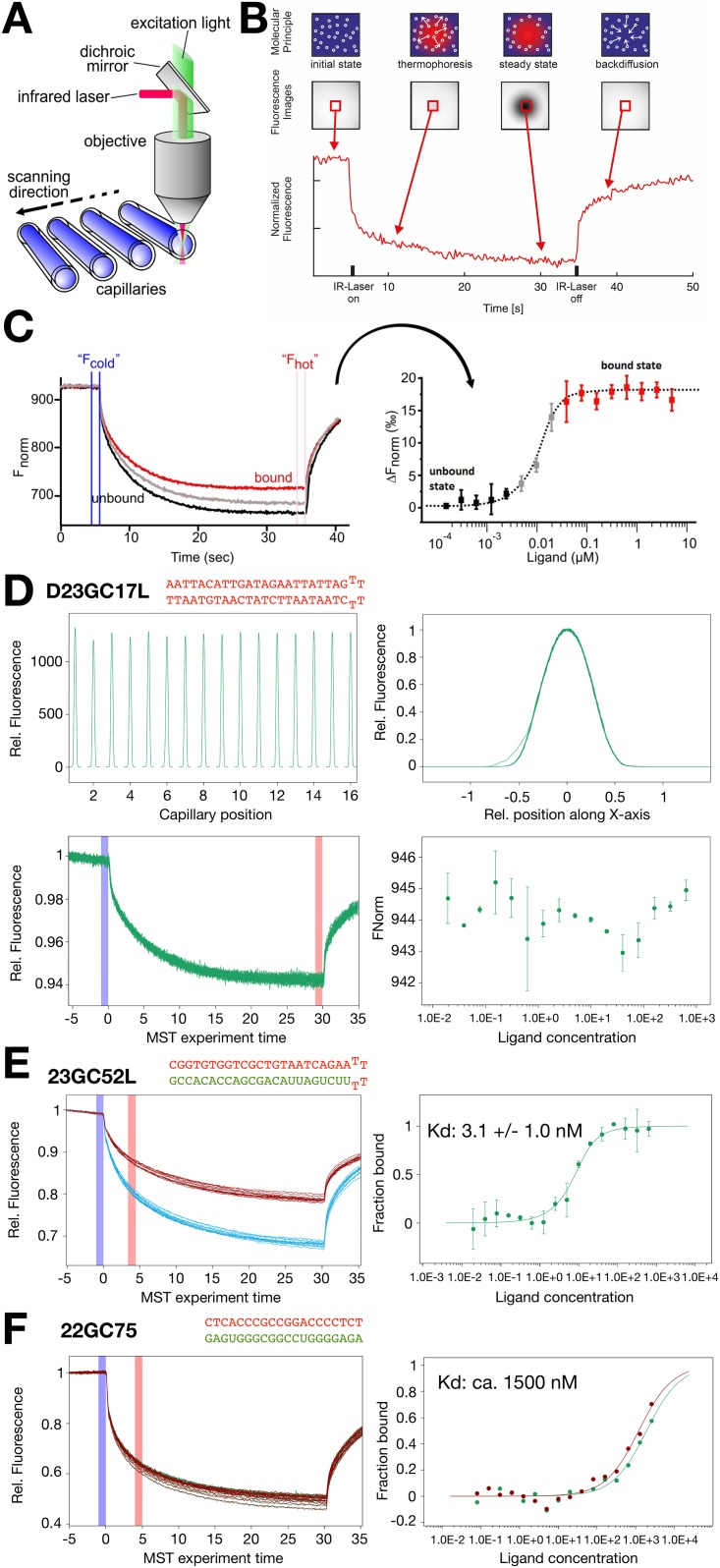
Determination of R-loop-antibody interactions by MST. **(A)** The Technical setup of the MST technology is shown. The optics focuses in the center of the glass capillaries, thereby detecting the fluorescence signal of the labelled molecule. An IR-laser is used to establish a temperature gradient in the observation window of the optical system. Changes in fluorescence intensity are used to monitor thermophoretic movement of the molecules in solution. The concentration of the R-loop sequence is kept constant in our assays and the concentration of the antibody was varied. **(B)** A single MST time trace, showing the changes in fluorescence due to the movement molecules in a temperature gradient. After an initial cold phase (5 sec, laser off), the laser is switched on and instantly establishes the temperature gradient. After the T-Jump phase, in which the fluorescent dye decreases its signal yield due to heat induction, the thermophoretic movement starts. After 30 sec the IR-laser is turned off and the molecules diffuse back. **(C)** Interpretation of the results of a typical MST experiment. The MST time traces of 16 capillaries containing the same concentration of fluorescently labelled R-loop and an increasing concentration of the unlabelled S9.6 antibody used in our study are recorded and plotted in one graph (left panel). The normalized fluorescence of the MST traces is plotted against the concentration of the ligand (right panel). The data points are fitted to obtain the binding affinity. **(D)** MST data analysis of the double stranded DNA oligonucleotide D23GC17L, serving as a no binding control. The top-left panel shows the capillary scan to monitor potential sticking effects and absolute fluorescence signals. The overlay of the 16 capillary scan reveals a homogeneous curve shape, indicating no sticking (top-right). The bottom-left panel shows an overlay of 16 the recorded, normalized thermophoresis curves. As the DNA is not expected to bind the curves do perfectly overlap over the antibody concentration range of 640nM to 20pM. The bottom-right plot shows the normalized fluorescence Fnorm (‰) from T-Jump and Thermophoresis vs. the concentration of antibody. STDEV derives from two repeats. The signal does not significantly change, indicating no binding. **(E)** MST data analysis of the oligonucleotide 23GC52L, forming an R-loop. Two individual experiments performed at different MST power conditions (20% and 40%), creating a temperature gradient of either about 1.5°C or 3°C, are plotted (left panel). The right plot shows fraction bound calculation and the corresponding K_d_ fit. **(F)** MST data analysis of the oligonucleotide 22GC75, forming an R-loop. One set of thermophoresis curves of three independent experiments is plotted (left panel). The calculated fraction bound of two experiments is plotted, showing that full binding is not achieved. The binding affinity is estimated from the binding curves and given.

The binding reaction is evaluated in 16 parallel reactions, containing a fixed concentration of the fluorescently labelled R-loop, incubated with a serial dilution of the S9.6 antibody in a final volume of 10ul. About 4–6 μl of each reaction is loaded into the glass capillaries and processed serially by the NanoTemper instrument. The infrared laser establishes a temperature difference ΔT of 2–6°C, depending on the MST power applied (20–80%) (Fig 1A). Fig 1B displays a typical MST experiment. During the first 5 seconds of the experiment, sample homogeneity is evaluated by monitoring a stable baseline of the fluorescence (“initial state”). Then, the IR-laser is switched on, causing an initial steep drop of the fluorescence signal—the so-called Temperature- or T-Jump—reflecting changes in the quantum yield of the fluorophore. Following the T-Jump the slow, thermophoresis-driven depletion of fluorophores occurs takes place. Once the infrared laser is deactivated, a reverse T-Jump and concomitant backdiffusion of the fluorescently labelled molecules can be observed. Binding parameters of a molecular interaction can be determined by MST, since thermophoretic properties correlate with the molecular properties such as size, hydration shell and charge. In a MST experiment, the serial dilution of the ligand and the concentration dependent ratios of bound and non-bound molecules give rise to specific thermophoresis curves, as shown in Fig 1C. Normalized bound and non-bound thermophoresis curves, as well as the partially bound intermediate curves are plotted, allowing the quantitative evaluation of the binding parameters (Fig 1C, right panel).

First, we tested as negative control the looped DNA oligonucleotide (D23GC17L) and measured its binding to the S9.6 antibody. An important quality control for MST is the so called capillary scan revealing the absolute amount of fluorescently labelled probe per capillary and allowing to monitor sticking effects. In our case, the plotting of all capillary scans in one graph revealed homogeneous capillary shapes and no sticking of the fluorophore to the glass walls (top panels in Fig 1D). The 16 recorded thermophoresis curves plotted in the graph (lower, right panel) were measured with 40% MST power, resulting in a ΔT of about 3°C. The highly overlapping thermophoresis curves show no significant changes in the curve shape with decreasing antibody concentrations, indicating no binding at these conditions (S9.6 Antibody concentration range: 1066nM to 16pM). Indeed, the quantitative evaluation of the normalized fluorescence over the ligand concentration reveals no significant fluorescence intensity changes and therefore no binding. Next we used as a positive control an R-loop that was previously characterized by Phillips and colleagues (23GC52L), revealing a high binding affinity of 0.47 nM in SPR assays [15]. The binding of 23GC52L (1nM) was measured with a decreasing S9.6 antibody concentration (1066nM to 16pM) using the NanoTemper NT.115pico system harbouring high sensitivity fluorescence detectors, allowing to determine binding constants in the picomolar range. Measurements performed at 20, 40 and 80% MST power (only the thermophoresis curves at 20 and 40% MST power are plotted in Fig 1E) revealed qualitative differences in curve shapes with decreasing antibody concentrations. Plotting of the normalized fluorescence over the antibody concentration and including the values of the 2 biological and 2 technical repeats into the graph, revealed a clear binding curve. Quantification of the data provides a K_d_ of 3.1 (± 1.0) nM, being almost an order of magnitude higher than the K_d_ determined by SPR. This discrepancy can be attributed to a known feature of SPR instruments using surface coupled assays, which are prone to reveal lower binding affinities due to surface sticking/avidity effects [32–34]. Still, we confirm the data of Phillips and colleagues, showing that the S9.6 antibody does bind with high affinity to this R-loop sequence.

Next, we tested a sequence with higher GC content (22GC75), lacking the T-loop that links both strands of the oligonucleotides to ease the formation of the double stranded R-loop. Using the same conditions as described above, we did not detect quantitative binding of the S9.6 antibody to this R-loop sequence. Fig 1F shows as an example the thermophoresis curves recorded at 40% MST power and the plot of the normalized fluorescence over the antibody concentration. The binding curves (20% and 40% MST power) of individual replicates show a similar behaviour, but do not reach a plateau that would indicate full binding. Therefore, binding is indicated with a calculated binding affinity of about 1500nM.

The lack of high affinity binding may be explained either by the recognition of the T-loop in the structure of 23CG52L by the antibody that that is missing in 22GC75, or by sequence preference in Antibody binding. The role of the T-loop in S9.6 binding was not evaluated by Phillips and colleagues, but as we describe below antibody binding is independent of the T-loop, suggesting sequence specificity in antibody binding.

**Fig 2:**
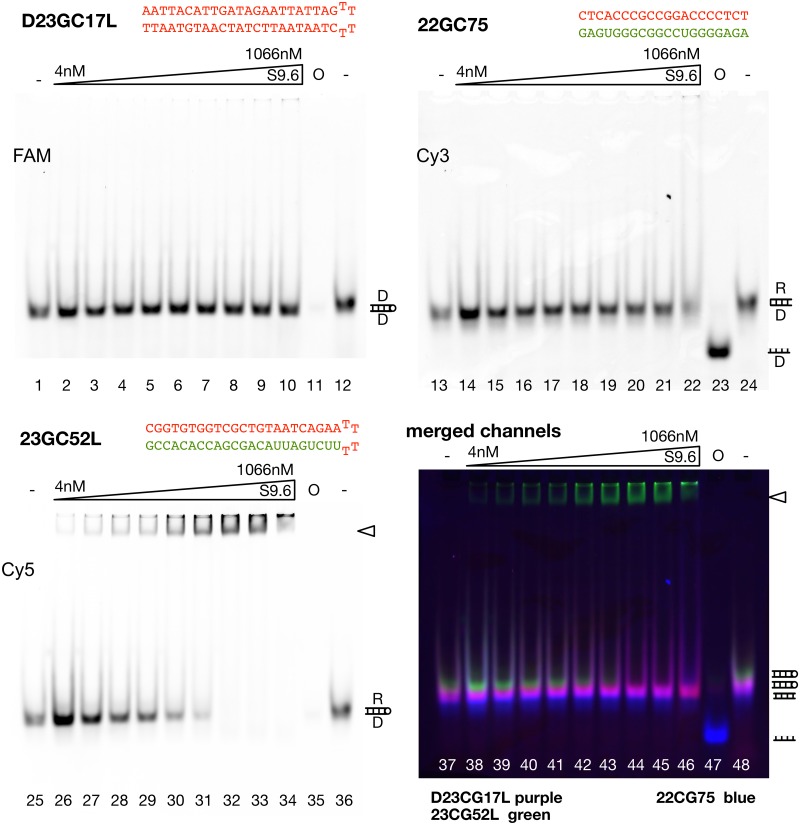
Analysis of R-loop-antibody-interaction by electromobility shift assays. To directly compare the binding properties of different R-loops and to provide internal controls for the assay we use three different fluorescent labels. Oligonucleotides are either labelled with Cy5, Cy3 or FAM dyes, mixed in stoichiometric amounts and incubated with increasing concentrations of the S9.6 antibody. The channels of the fluorescence scans are given individually and as a merge (bottom-left panel). The R-loops and the DNA control used in Fig 1 were tested in EMSA. The mixture of substrates was incubated for 30 min with the increasing concentrations of S9.6 antibody (4nM to 1066nM) as indicated. Samples were separated on 8% native polyacrylamide gels. Lane 23 (47) reveals the migration behaviour of the single-stranded DNA (O: Oligonucleotide) for the R-loop substrate that was prepared from two individual molecules (22GC75). The triangle marks nucleic acid-antibody complexes. The positions of DNA and R-loop substrates is indicated on the right of the gel (D: DNA strand; R: RNA strand).

To have an additional method to prove the specificity and efficiency of S9.6 binding to R-loops, we established the multicolour electromobility shift assays (EMSA). Here we combine two or three different fluorescently labelled nucleic acid probes in one test tube and incubate them with increasing S9.6 antibody concentrations. R-loop-antibody reactions are separated on native polyacrylamide gels and relative binding affinities can be evaluated in this competitive binding assay (Fig 2). In the experiment shown in Fig 2 we mixed the Cy3, Cy5 and FAM labelled double stranded DNA D23GC17L and the R-loops 23GC52L and 22GC75 used in the first MST assay described above. As shown in the MST experiments, the antibody does not bind to double stranded DNA in the electromobility shift assay (panel: D23GC17L). However, a specific electromobility shift product is detected with the R-loop 23GC52L (Fig 2, indicated by a triangle) that is incubated with the double stranded DNA in the same reaction setup. In contrast, the R-loop 22GC75 exhibiting similar size, but higher GC content, shows only partial binding at the highest antibody concentration, like in the MST experiment. The EMSA experiment confirms the MST result in that the antibody S9.6 displays different binding affinities between R-loop sequences. Evaluating the binding affinity from EMSA gels, measuring the concentration of half maximum binding of the free R-loop reveals a binding affinity of about 10nM for 23GC52L and a binding affinity of about 1000nM for 22GC75. Deviations between MST and EMSA may result from the different buffer properties and caging effects of the gel based assay system.

**Fig 3:**
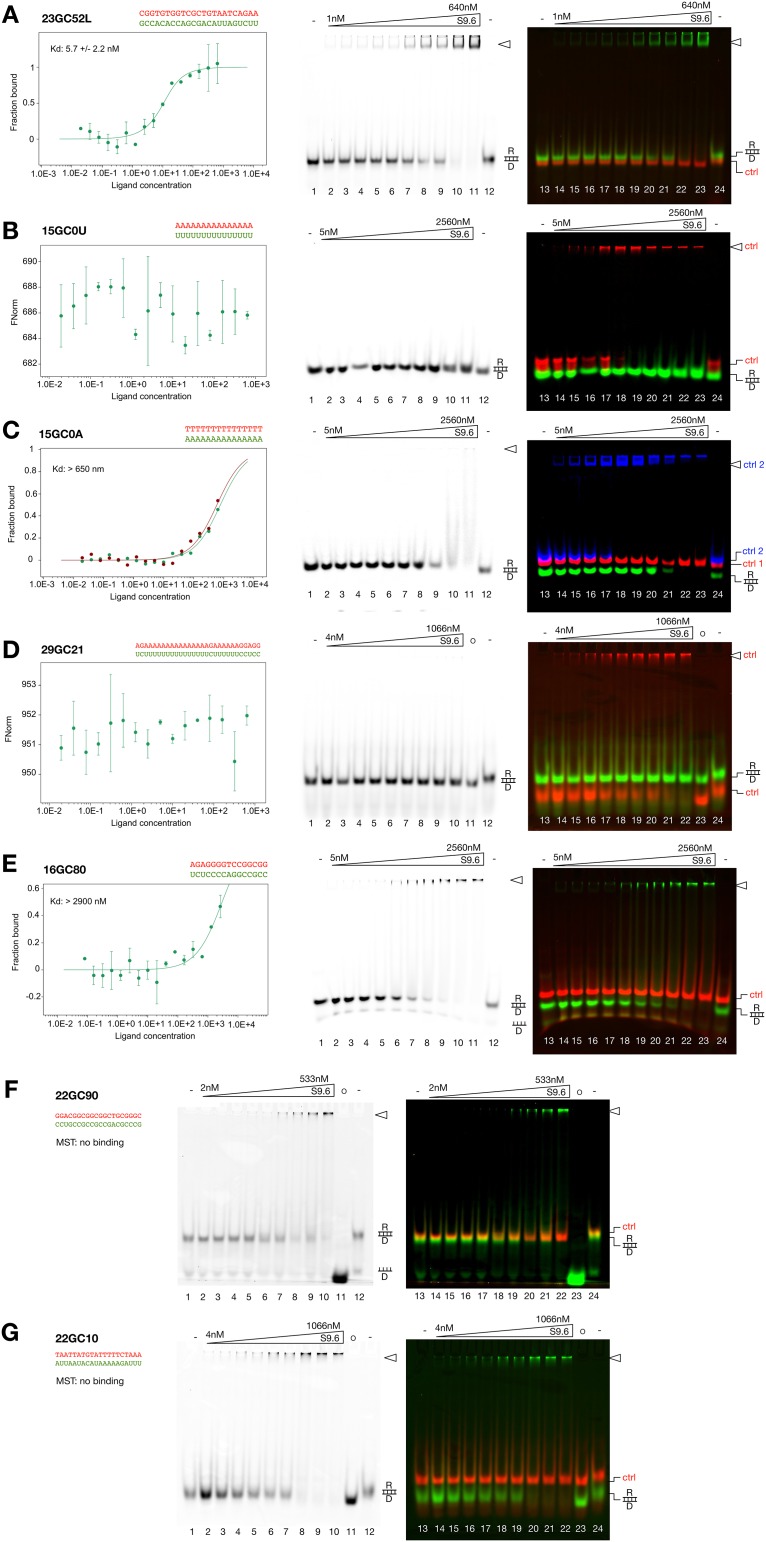
Summary of the EMSA and MST results. The respective MST binding curves are plotted on the left, the fluorescence channel depicting the EMSA of R-loop substrate is shown in the middle panel and the merge of the EMSA fluorescence channels revealing the substrate and the controls is shown on the right. The concentration of S9.6 antibody used for the individual experiments is shown on top of the gel. The nucleic acid-antibody complexes are indicated by a triangle and control R-loops (ctrl1 and ctrl2) are indicated on the right side of the gel. **(A)** Analysis of the R-loop 23GC52. The double stranded DNA D23GC17L served as negative control (ctrl1, red). **(B)** Analysis of the R-loop 15GC0U. The R-loop 23GC52L served as positive control (ctrl, red). **(C)** Analysis of the R-loop 15GC0A. The double stranded DNA D23GC17L (ctrl1, red) served as negative control and the R-loop 23GC52L (ctrl2, blue) served as positive control. **(D)** Analysis of the R-loop 29GC21. The R-loop 15GC0A (ctrl, red) served as positive control. **(E)** Analysis of the R-loop 16GC80. The double stranded DNA D23GC17L (ctrl, red) served as negative control. **(F)** Analysis of the R-loop 22GC90. The double stranded DNA D23GC17L (ctrl, red) served as negative control. **(G)** Analysis of the R-loop 22GC10. The double stranded DNA D23GC17L (ctrl, red) served as negative control.

To further address the sequence specific features of the S9.6 antibody we measured side by side binding affinities of the R-loops shown in Table 1, by MST and EMSA. To rule out the recognition of the T-loop by the antibody, we only used sets of two linear oligonucleotides to form the R-loops (Fig 3A). 23GC52 has the same sequence as the high affinity binder 23GC52L, but lacking the T-loop. This R-loop without T-loop displays a binding affinity of 5.7nM (±2.2) in MST and a correspondingly weaker binding affinity in EMSA (about 40nM). This experiment reveals that the T-loop has a neglectable effect on the overall binding affinity of the antibody.

Then we used a variety of other nucleic acid sequences in the length range of 15 to 29bp and a GC-content from 0 to 90%. The minimal R-loop binding length determined by Phillips and colleagues was 6bp [15], being much below the R-loop sequences used for our study. Surprisingly, all the R-loops showed a much reduced, or non-detectable binding affinity in MST and EMSA experiments. Due to our internal controls used in the EMSA we can assure that it is not an experimental problem, but obviously the individual R-loop sequences bind with highly variable affinities to the antibody.

Using an R-loop with 100% AU-sequence (15GC0U), meaning that the RNA strand contains only Uridines, exhibits no binding in MST, nor in EMSA (Fig 3B). In contrast, having only Adenines in the RNA strand and Thymine in the DNA strand reveals weak binding (15GC0A; Fig 3C). In MST the R-loop 15GC0A reveals indicated binding with a Kd of >700nM and in EMSA we estimated a Kd of about 1200nM.

These differences are not depending on the short length of the oligonucleotides, even if we measure a 29bp long R-loop and increase the GC content to 21%, flanking a 15 bp long U-rich region (29GC21), we do not observe binding in MST nor in EMSA (Fig 3D). Next we increased the GC-content in the R-loop sequences to test whether this would determine high affinity binding, since R-loops are predominantly formed at GC-rich genomic regions [35]. We have shown no binding or weak binding to sequences in the range from 0 to 25% CG content, identified strong binding to sequence with 52% CG content and weak binding to a sequence with 75% GC content (22GC75). Two more sequences with 80% (16GC80) and 90% (22GC90) GC-content were tested, revealing weak or no binding in MST and weak binding in MST (Fig 3E and 3F), suggesting that the binding affinity strongly depends on DNA sequence and cannot be compensated by rather longer R-loop sequences of up to 29 bp in sequence length.

Having a GC-content of 10% distributed throughout the R-loop sequence of 22bp (22GC10, Fig 3G) showed specific binding in EMSA, with an apparent binding affinity of about 100nM, but no quantifiable binding in MST. MST and EMSA strongly deviate in several of the R-loop constructs, especially with complexes binding with rather lower affinity in EMSA, or forming untypical shifts in EMSA that do not enter the gel. The untypical shifts suggest two modes of binding. Either the complexes form discrete bands in EMSA, then they have a high affinity in EMSA and MST, or they do not enter the gel, as if larger, non-specific precipitates would form in EMSA. In case of the EMSA precipitates we cannot observe clear MST signals, rather suggested no binding in these cases.

## Discussion

The S9.6 antibody is an important tool in studying RNA-DNA hybrids, but already a study performed about 27 years ago suggested limitations of this tool, as this antibody was successfully used to detect RNA-RNA duplexes [36]. A recent study then quantified the binding affinity towards double stranded RNA showing that S9.6 binds with an Kd of 2.7nM to the AU-rich sequence [37]. Accordingly, it is now suggested to include an RNase A preclearing step prior to the immunoprecipitation reaction, in order to ensure the specific precipitation of R-loop sequences [38]. However, since the structure of dsRNA and R-loops do differ significantly, it is unlikely that the antibody recognizes solely the phosphate-sugar backbone structure of the R-loops, which would be required for sequence independent binding [28]. Similarly, we observe that dsDNA, also exhibiting a distinct structure with respect to dsRNA and R-loops, is not recognised by the antibody, suggesting that the binding motifs are embedded in defined structural contexts. Therefore, we assume that antibody binding is determined by a combination of sequence and additional structural constraints. Indeed, our experiments clearly show a strong dependency of S9.6 binding on the sequence of the R-loop in EMSA and MST experiments. The absolute binding parameters partly deviate in MST and EMSA, but this may be explained by the different buffer conditions and the “caging effects” of the EMSA and probably different binding modes of the antibody with the R-loop sequences. These include the “matrix interaction effects”, “excluded volume effects” and “solvation effects” that may differently affect nucleic acid-antibody interactions of different binding affinity [39].

Essentially we could reproduce the binding affinity of the 23GC52L R-loop, previously measured by Phillips and colleagues [15], showing that we use the correct binding conditions. However, by varying the sequence context of the R-loops and the GC-content we were not able to detect high affinity binding any more. Both assays MST and EMSA did convincingly reveal a broad spectrum of binding affinities that do not correlate with GC-content of the R-loop sequence. Essentially our studies suggest that immunoprecipitation-experiments, after RNase A treatment, could result in an underestimation of R-loop regions throughout the genome, especially if short R-loop stretches are studied. Still, one should mention that short R-loop sequences in cells are unlikely to be stable and that the antibody may pull down longer R-loop domains with high specificity. The bias in R-loop sequence annotation must be considered in future studies and better tools are required to study R-loop formation *in vivo*.
